# The perverse fisheries consequences of mosquito net malaria prophylaxis in East Africa

**DOI:** 10.1007/s13280-019-01280-0

**Published:** 2019-11-11

**Authors:** Benjamin L. Jones, Richard K. F. Unsworth

**Affiliations:** 1 Department of Ecology, Environment and Plant Sciences, Stockholm University, 106 91 Stockholm, Sweden; 2 Project Seagrass, 33 Park Place, Cardiff, CF10 3BA UK; 3 Seagrass Ecosystem Research Group, College of Science, Swansea University, Wallace Building, Swansea, SA2 8PP UK

**Keywords:** Bed net fishing, Landing survey, Mosquito net fisheries, Poverty, Seagrass fisheries

## Abstract

Malaria is a serious global health issue, with around 200 million cases per year. As such, great effort has been put into the mass distribution of bed nets as a means of prophylaxis within Africa. Distributed mosquito nets are intended to be used for malaria protection, yet increasing evidence suggests that fishing is a primary use for these nets, providing fresh concerns for already stressed coastal ecosystems. While research documents the scale of mosquito net fisheries globally, no quantitative analysis of their landings exists. The effects of these fisheries on the wider ecosystem assemblages have not previously been examined. In this study, we present the first detailed analysis of the sustainability of these fisheries by examining the diversity, age class, trophic structure and magnitude of biomass removal. Dragnet landings, one of two gear types in which mosquito nets can be utilised, were recorded across ten sites in northern Mozambique where the use of Mosquito nets for fishing is common. Our results indicate a substantial removal of juveniles from coastal seagrass meadows, many of which are commercially important in the region or play important ecological roles. We conclude that the use of mosquito nets for fishing may contribute to food insecurity, greater poverty and the loss of ecosystem functioning.

## Introduction

Malaria is arguably one of the most significant global health issues of recent times. With around 200 million cases, and 600 000 deaths per year globally (Lover et al. 2011; WHO 2016), non-governmental organisations, foundations, trusts and philanthropists have invested millions in the mass distribution of mosquito nets as a means of potential prevention. As such, large scale initiatives such as the RollBack Malaria Programme (www.endmalaria.org) have distributed hundreds of millions of nets globally (Lover et al. 2011). While these nets are intended to be used for malaria protection, many have been repurposed for alternative uses, ranging from covering crops to making clothes, such as wedding dresses (Eisele et al. 2011; Lover et al. 2011). However, increasing evidence now suggests fishing is a primary use of mosquito nets (Short et al. 2018), providing new concerns for the already stressed coastal environments of the tropics (Valiela et al. 2001; Bellwood et al. 2004; Waycott et al. 2009).

Innovation and opportunism in small-scale fisheries is crucial to communities seeking to rise to the challenges of increasing human population, changing governance or climatic drivers (Locke et al. 2017). In cases, this can result in higher rates of catch, but generally only for a limited time. For example, by switching from bamboo-based fish-traps to net traps, which are easier to deploy, more efficient, much lighter and last longer, fishing communities in the Solomon Islands inadvertently caused an increase in fishing effort that led to a decline in fish stocks (Locke et al. 2017). The use of mesh mosquito nets as fishing gear in shallow water environments demonstrates this opportunism (Short et al. 2018), where their strength, light weight and free or cheap accessibility make them an attractive tool for fishing. The use of mosquito nets for fishing is now common place across the globe, potentially occurring in over 30 countries, half of which are in Sub-Saharan Africa (Short et al. 2018). Observations of such use are highest within the Western Indian Ocean, but to date, as far as the authors are aware no quantitative data exist on their actual landings. With potentially 150 million mosquito nets distributed yearly (WHO 2016), the use of such nets for fishing is likely to continue despite bans, but whether this will be in detriment to the communities that use them is unknown.

**Fig. 1:**
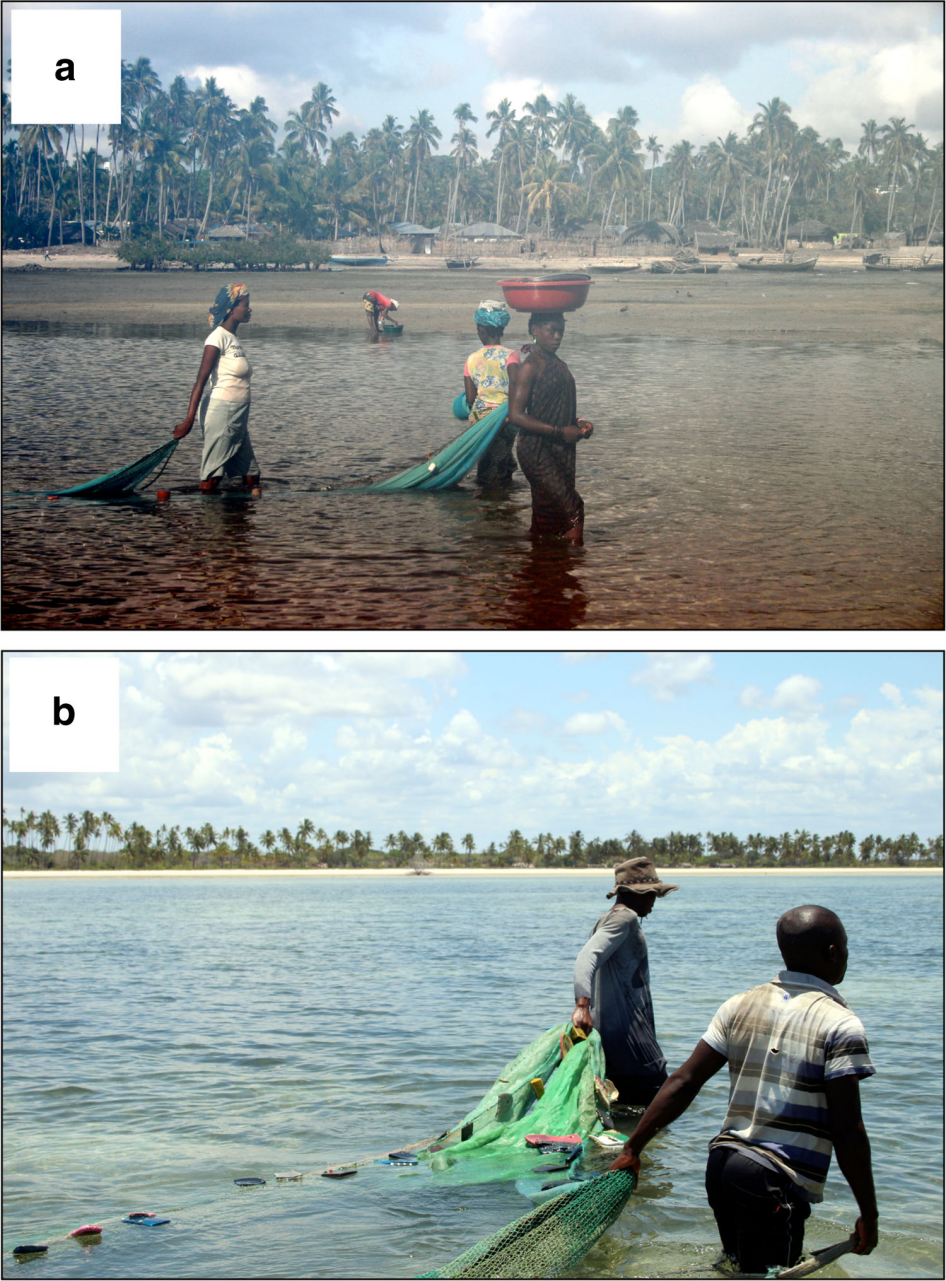
Mosquito nets can be utilised in fisheries in two ways: either as small beach seines, used by women or children (**a**), or forming part of larger drag nets used by men operating in teams of two or more (**b** photos: author provided)

Mosquito nets are generally used as artisanal fishing gear in two forms. The most common is as smaller lightweight seine nets which are usually utilised by women and children (Fig. 1a; author observations). However, in northern Mozambique, mosquito nets form parts of drag nets, used by men operating in teams of two or more from a canoe (Fig. 1b; author observations) (Gough et al. 2009). In some parts of the tropics filter nets are utilised similarly to mosquito nets however the filter nets tend to only have fine mesh towards the narrow end of a their nets (Cod end) (Santander and Monteclaro 2018).

Tropical small-scale and artisanal fishers depend on fish for both food security and livelihoods and use a range of gear types to collect fish. However, with depleting fisheries and increasing competition, artisanal fishers frequently resort to less selective methods to ensure that landings are sufficient for food supply or income (Jones et al. 2018). The use of mosquito nets is a potential example of this, with consequential negative implications for human health, fisheries sustainability and ecosystem resilience. Given that mosquito nets are available either for free or at minimal cost, it provides opportunities for all members of society to efficiently harvest resources from the marine environment with minimal effort, as expensive fishing gears and vessels are no longer required (Bush et al. 2017). With new entrants to the fishery, higher pressure is put on marine resources. The concern of mosquito net fisheries (MNF) however, is their small mesh size (generally ≤ 3 mm) and the coastal habitats in which they are used. Although there is a growing depth of literature describing the presence of MNF around the world (Short et al. 2018) we are not aware of any quantitative analysis of the landings that marine MNF provide or information on the habitat preference for these nets.

Seagrass meadows are highly productive, occur in tropical shallow areas (easy access) around the world and are a soft bottom habitat, making them ideal areas for MNF Seagrass meadows form a crucial component of tropical marine seascapes, covering great intertidal and subtidal areas of the Indo-Pacific (Short et al. 2007). As a critical habitat for a diverse array of fish and invertebrate species (Unsworth et al. 2014), seagrass meadows provide food security and livelihoods for coastal communities across the Indo-Pacific region (de la Torre-Castro and Rönnbäck 2004; Nordlund et al. 2010; Cullen-Unsworth et al. 2014; Unsworth et al. 2014). Seagrass fisheries are of fundamental importance to coastal communities in emerging economies because they are shallow and close to shore (Nordlund et al. 2018; Unsworth et al. 2018). In many cases seagrasses are much more accessible than coral reefs, especially to the most impoverished fishers in society whom either operate on foot (e.g. gleaning) or in small canoes (Nordlund et al. 2010; de la Torre-Castro et al. 2014).

Seagrass meadows provide a nursery function (Mumby 2006; Campbell et al. 2011; Nagelkerken et al. 2013), and as such harbour diverse and abundant populations of juveniles. Mosquito nets potentially harvest a large proportion of these juvenile species (Bush et al. 2017; Short et al. 2018). While smaller species may provide vital nutrition to those in poverty (Kawarazuka and Béné 2011), the removal of juvenile species before they reach sexual maturity is a recipe for overfishing and already an issue in the Indo-Pacific region (Darkey and Turatsinze 2014). Both temperate and tropical seagrass ecosystems are defined by top-down predator control (Eklof et al. 2008a; Baden et al. 2012), and many juvenile and adult predatory species use seagrass meadows to feed and hide, thus removing these species can induce trophic cascades and threaten the sustainability of the resource (Pauly et al. 2005).

Despite a growing body of information on the ecological and economic importance of seagrass meadows globally, quantitative information is lacking that can help improve understanding of the consequences of overfishing in this ecosystem. The combined issues of MNF are of fundamental concern, and there exists an urgent need to categorise MNF catch from seagrass meadows, and more broadly. In this study, we present the first quantitative analysis of the catch composition and biomass of a drag net marine MNF and discuss the potential implications for local resource use, ecosystem structure and function.

## Materials and Methods

### Study location

**Fig. 2:**
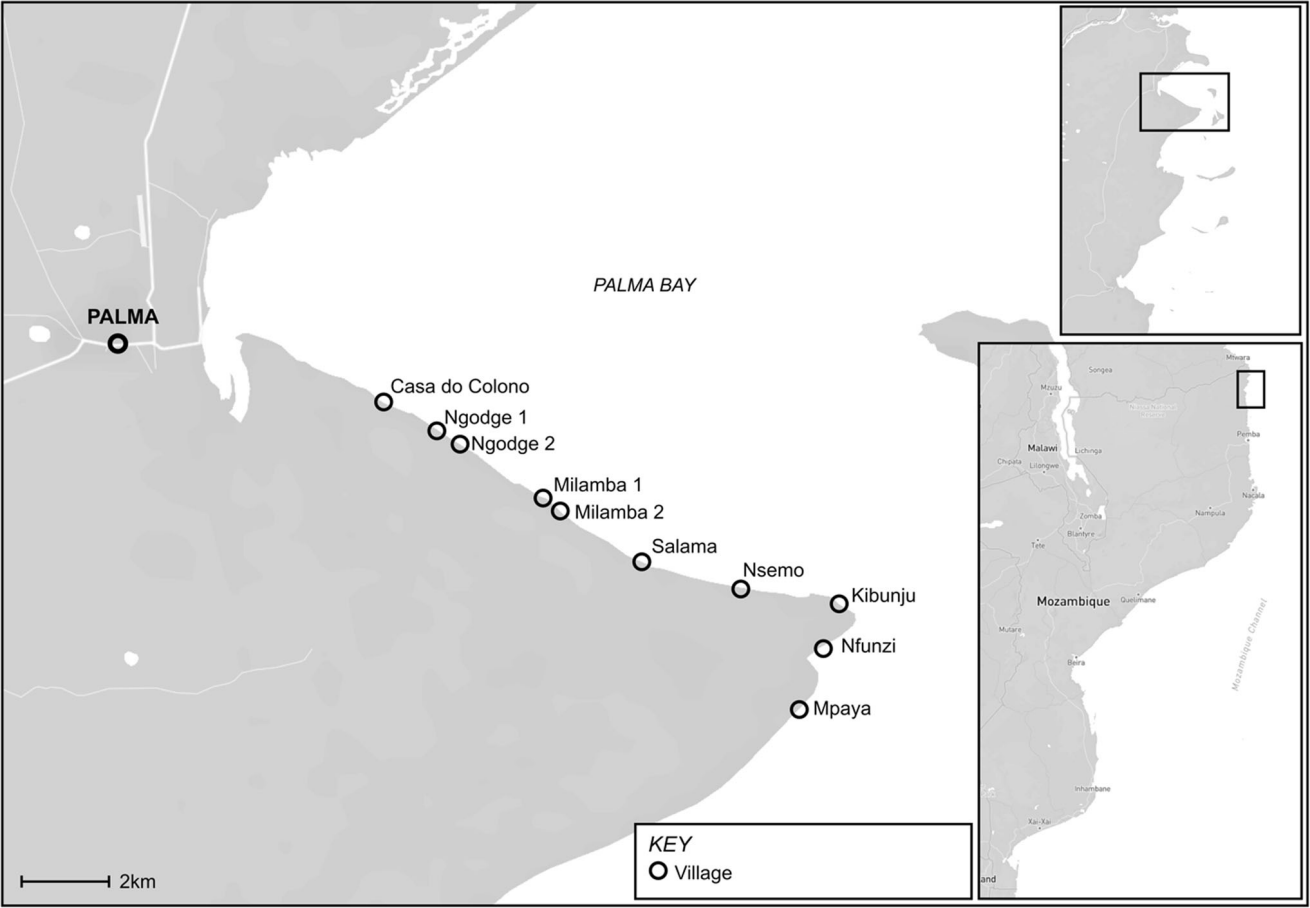
Map of Palma Bay, Cabo Delgado Province, Mozambique showing the location of the ten villages where mosquito net fishery catches were analysed

The present study took place in Palma Bay (S 10° 45′ 44.03″, E 40° 32′ 42.97″), situated in Palma District, Cabo Delgado Province in the north of Mozambique (Fig. 2); a region with a population of nearly 2.5 million people. Across the Cabo Delgado Province there are potentially more than 26 000 active fishers, none of which are officially recorded in Mozambique’s National Fisheries Statistics (Jacquet et al. 2010). Fisheries landing surveys were conducted in ten villages situated to the south of Palma bay. These were Casa do Colono, two sub-villages of Ngodge, two sub-villages of Milamba, Salama, Nsemo, Kibunju, Nfunzi and Mpaya. The research occurred with full permission from local government agencies and community groups. Except for their distance from Palma town (the population hub), and thus potential fishing intensity, all sites were characteristically similar. Mixed habitats occur within the bay, including coral reefs, mangroves, large sand banks and deep channels. Intertidal areas were characterised by mixed seagrass meadows dominated by *Cymodocea serrulata* and *Thalassia hemprichii* in shallow areas, shifting to *Enhalus aceroides* in subtidal areas and *Thalassodendron ciliatum* in deeper areas. The bay is sheltered from heavy seas by the islands of Tecomaji and Rongui but is subject to strong tidal currents. Weather patterns are dominated by large scale pressure systems of the western Indian Ocean, the dry northeast monsoon (October to March) and wet southeast monsoon seasons (April to September).

### Mosquito net fishery survey

Catch composition and biomass of mosquito net fishery (MNF) landings at each of the villages (see Fig. 1) were assessed over a ten-day period in 2014 (between October and November; one village per day). The same two fishers assisted the survey at all sites, using the same drag net at each site and fishing as they normally would. The drag net (22 × 1.5 m) consisted of a series of nets (mesh size < 5 cm), sewn together, with a finer mosquito net end bag/cod end, and was dragged parallel to shore by the fishers, working ~ 7.5 m apart, for 10 min wading on an incoming tide. All drags were conducted over seagrass areas (dominated by *Halodule uninervis*, *Halophila ovalis*, *Cymodocea* spp. and *Thalassia hemprichii*), which were randomly chosen by the two fishers. In all instances, the first drag at each site was conducted 1 h after low tide (to allow the water to rise above the seagrass slightly). This fishing method is characteristic of drag net MNF activity across the region (Gough et al. 2009). GPS co-ordinates were taken at the start and end of each deployment to measure drag distance in order to calculate swept area.

**Fig. 3:**
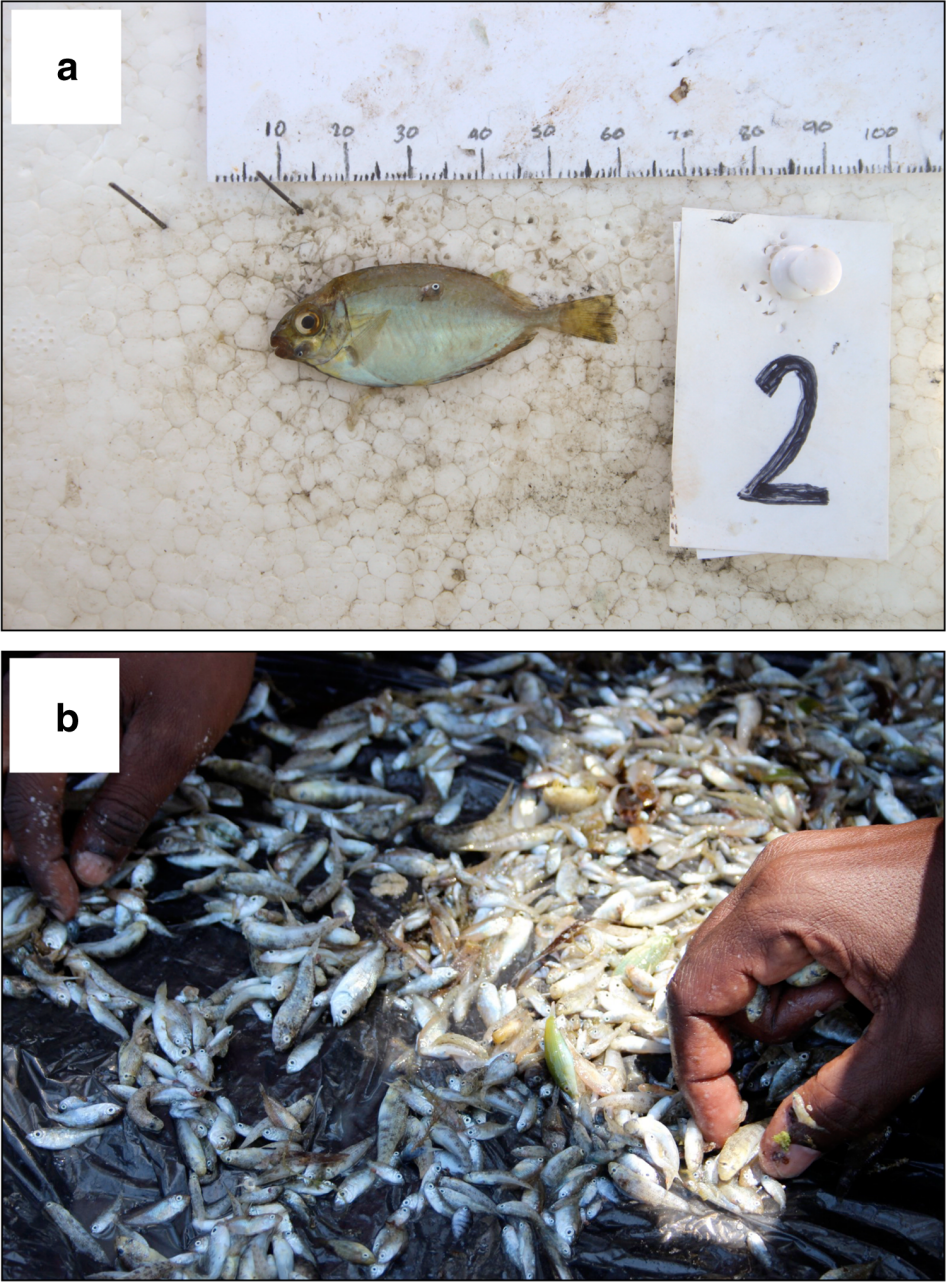
Representative size of *Siganus* sp. (**a**) and wider assemblage (**b**) caught in seagrass meadows using a drag net in Palma Bay, Cabo Delgado Province, Mozambique (photos: author provided)

A total of 25 drags were conducted; three drags each at Casa do Colono, Ngodge 1, Salama, Nfunzi and Mpaya, and two drags each at Ngodge 2, Milamba 1, Milamba 2, Nsemo and Kibunju. After each drag, catch was retrieved and placed in buckets stored within a canoe for later identification and sorting, before being given back to the fishers for sale or consumption. All species were retained and photos taken to assist with species identification. Upon sorting the catch, total catch weight was recorded, as was the weight of each individual species group. We estimated the size of individual species using representative individuals that reflected most of the catch (Fig. 3a), given that recording the quantity and sizes of all and even a subset of individuals was deemed too difficult given the large quantity (< 1000) and small size of most individuals (< 20 mm; Fig. 3b). To determine the juvenile composition of the catch, size at maturity data was collated from the FishBase (Froese and Pauly 2016). Where length at maturity data was not available it was defined by one-third of the maximum length of each species (Harmelin-Vivien et al. 1985). From the field estimations described above, species sampled were then placed into one of three categories; (a) Present in samples as juveniles only, (b) Present in samples at all life-history stages or (c) Present in samples as adults only. The trophic roles and commercial importance of each of the species recorded were also collated from FishBase (Froese and Pauly 2016).

### Data analysis

Data, where presented is reported at mean ± SD. The swept area (*a*, m^2^) of each drag was calculated as:

$$ a = D \times h \times X $$

where *D* is total drag distance (m), *h* is the head-rope length (m) and *X* the fraction of head-rope length equal to the swept path-width, set at 0.5 (Pauly 1980).

Catch-per-unit area was (CPUA, kg m^−2^) for each drag was calculated as:

$$ {\text{CPUA}} = \frac{C/t}{a/t} $$

where catch *C* is the total weight of each catch (kg) and *t* is the time spent conducting the drag (h).

## Results

### Mosquito net survey

In total, 39 species from 26 families were recorded in mosquito net fishery (MNF) catches from coastal seagrass meadows of Palma Bay (Table 1). A total of 25 drags were conducted, with a mean distance of 82.4 ± 26.1 m and mean swept area of 809.5 ± 393.2 m^2^. The mean drag biomass was 1.4 ± 1.5 kg and CPUA was 0.017 ± 0.0017 kg m^−2^. On average, each drag landed 10.96 ± 2.75 species.

**Table 1:** Species recorded in mosquito net fishery catches from seagrass meadows in Palma Bay, Mozambique, categorised on the notion that they were either present as juveniles (J), present at all life-history stages (O) or present as adults (A). Species were categorised based on representative length and length at maturity data was obtained from Froese and Pauly (2016) or calculated where not available. Commercial value is noted by an asterisk next to species name where * = low, ** = medium, *** = high and **** = very high and were obtained from Froese and Pauly (2016) along with Trophic Level, also obtained from Froese and Pauly (2016). Areas of grey shading represent data that are unavailable

Family	Species	Common name	J	O	A	Trophic group	Trophic level
Finfish							
Albulidae	*Albula oligolepis*	Smallscale bonefish	x			Invertivore	3.3 ± 0.2
Apogonidae	*Foa brachygramma*	Weed cardinalfish			x		3.5 ± 0.5
	*Fowleria variegata*	Variegated cardinalfish			x		3.5 ± 0.5
Balistidae	Sufflamen chrysopterum**	Halfmoon triggerfish	x			Invertivore	3.5 ± 0.41
Bleniidae	*Petroscirtes mitratus*	Floral blenny					2.2 ± 0.0
Bothidae	Bothus pantherinus****	Leopard flounder	x			Invertivore	3.5 ± 0.37
Callionymidae		Dragonet sp.					
Clupeidae	Sardinella gibbosa*	Goldstripe sardine	x			Planktonivore	2.9 ± 0.30
Fistulariidae	Fistularia commersonii*	Bluespotted cornetfish	x			Inverts and fish	4.3 ± 0.7
Gerreidae	Gerres oyena**	Common silver-biddy	x			Invertivore	2.7 ± 0.24
Gobiidae	*Amblygobius albimaculatus*	Butterfly goby		x		Invertivore	2.6 ± 0.2
	*Asterropteryx ensifera*	Miller’s damsel			x	Zooplanktonivore	3.4 ± 0.45
	*Exyrias belissimus*	Mud Reef-goby			x	Invertivore	2.8 ± 0.30
	*Gnatholepis cauerensis*	Eyebar goby			x	Omnivore	2.3 ± 0.0
	*Oplopomus oplopomus*	Spinecheek goby			x		3.4 ± 0.3
Haemulidae	Plectorhinchus gaterinus**	Blackspotted rubberlip	x			Inverts and fish	4.0 ± 0.66
Labridae	Cheilio inermis****	Cigar wrasse	x			Invertivore	3.5 ± 0.54
	*Cymolutes praetextatus*	Knife razorfish			x	Invertivore	3.6 ± 0.6
	Halichoeres scapularis****	Zigzag wrasse	x			Invertivore	3.5 ± 0.50
	Stethojulis bandanensis****	Red shoulder wrasse			x	Invertivore	3.2 ± 0.2
	*Stethojulis* *strigiventer*	Three-ribbon wrasse			x	Invertivore	3.1 ± 0.1
Lethrinidae	Lethrinus harak****	Thumbprint emperor	x			Inverts and fish	3.6 ± 0.5
Lutjanidae	Lutjanus ehrenbergii***	Blackspot snapper	x			Inverts and fish	3.9 ± 0.6
Ostraciidae	Ostracion cubicus***	Yellow box-fish	x			Omnivore	3.4 ± 0.48
Pempheridae	*Pempheris mangula*	Black-edged sweeper	x				3.4 ± 0.5
Pomacentridae	*Abudefduf sexfasciatus*	Scissortail sergeant	x			Planktonivore	2.7 ± 0.30
	*Abudefduf sparoides*	False-eye sergeant	x			Omnivore	3.0 ± 0.36
	*Abudefduf vaigiensis*	Indo-Pacific sergeant	x			Omnivore	2.6 ± 0.4
	*Chrysiptera annulata*	Footballer demoiselle	x			Omnivore	2.8 ± 0.31
Scaridae	Calotomus spinidens***	Spinytooth parrotfish	x			Herbivore	2.0 ± 0.0
	Leptoscarus vaigiensis***	Marbled parrotfish	x			Herbivore	2.0 ± 0.0
Serranidae	Epinephelus fuscoguttatus****	Brown-marbled grouper	x			Inverts and fish	4.1 ± 0.72
Siganidae	Siganus lurdis***	Dusky spinefoot	x			Herbivore	2.0 ± 0.0
Sphyraenidae	Sphyraena jello**	Pickhandle barracuda	x			Piscivore	4.5 ± 0.6
Syngnathidae		Pipefish spp.					
Synodontidae	Synodus jaculum**	Lighthouse lizardfish			x	Inverts and fish	4.0 ± 0.7
Tetraodontidae	Arothron hispidus****	White-spotted puffer	x			Omnivore	3.2 ± 0.0
Terapontidae	*Pelates quadrilineatus*	Fourlined terapon			x	Omnivore	3.6 ± 0.4
Invertebrate							
Penaeidae	*Fenneropenaeus indicus*	Indian prawn					

**Fig. 4:**
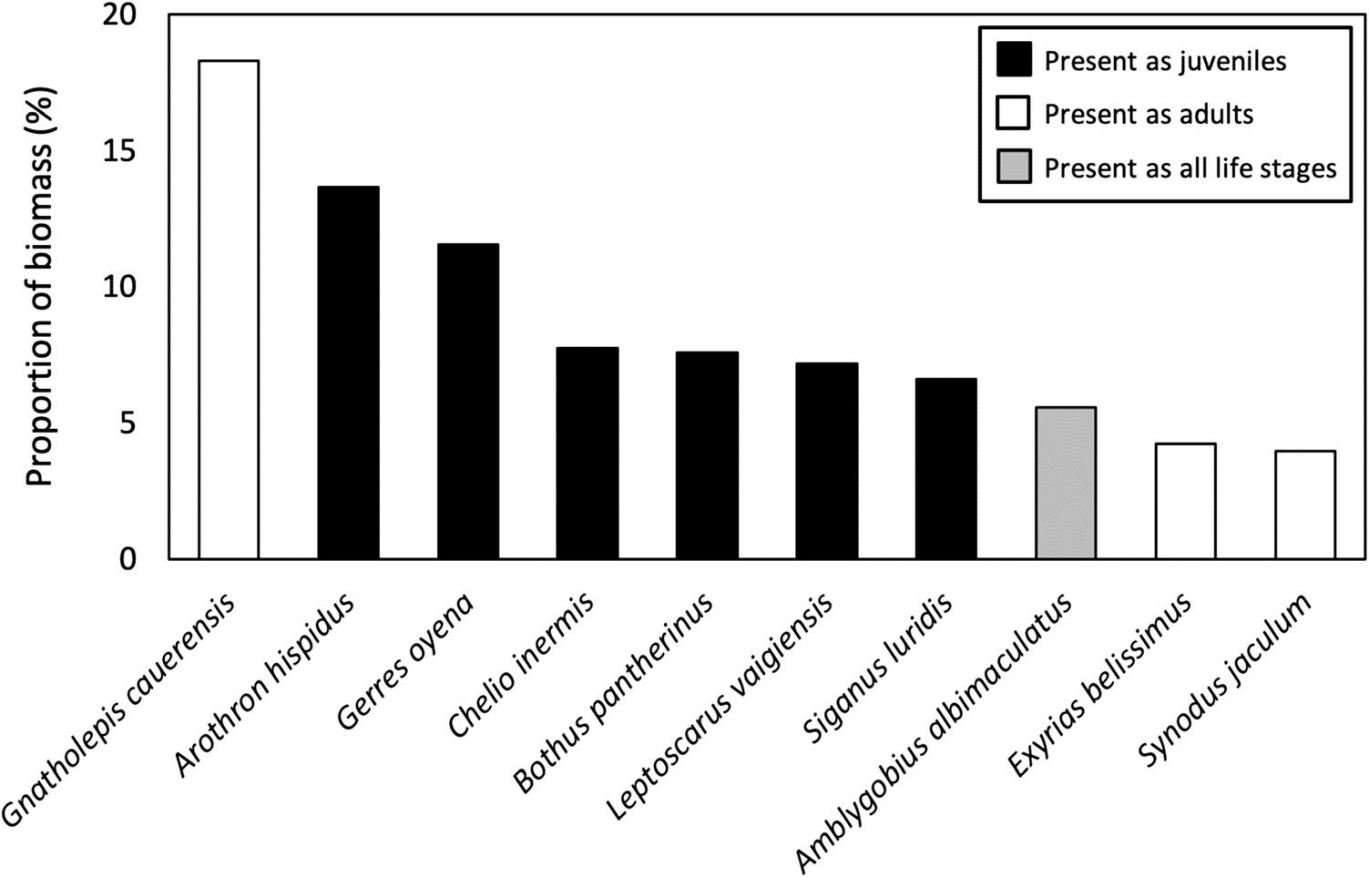
The ten most abundant fish species (percent of total weight) in drag net catches conducted over seagrass meadows in Palma Bay, Cabo Delgado Province, Mozambique. Species are grouped by whether they were present as juveniles (black), adults (white) or all life stages (grey)

Thirty-eight of the 39 species recorded were finfish with the remaining a species of invertebrate; *Fenneropenaeus indicus* (Indian prawn). The most abundant fish in terms of biomass were *Gnatholepis cauerensis* (Eyebar goby) with a total biomass of 6.59 kg. This was followed by *Arothron hispidus* (White-spotted puffer), *Gerres oyena* (Blacktip mojarra), *Chelio inermis* (Cigar wrasse), *Bothus pantherinus* (Leopard flounder), *Leptoscarus vaigiensis* (Marbled parrotfish) and *Siganus lurdis* (Dusky spinefoot); these species accounted for more than 70% of landed biomass (Fig. 4). In terms of frequency, *G. cauerensis*, *A. hispidus*, *G. oyena* and *S. lurdis* were most common, occurring in 84% of all catches.

### Life-history stage

Of the species sampled in this survey, 56% were classed as juvenile, 5% as all life stages and 26% as an adult (the remainder could not be categorised into age class). Of the ten most abundant species in terms of biomass, six were classed as a juvenile. Similarly, 73% of the species present as juveniles were noted as having importance either for commerce or subsistence including *G. oyena*, *Lutjanus ehrenbergii*, *Epinephelus fuscoguttatus*, *Sardinella gibbosa*, *Lethrinus harak*, *S. lurdis* and *L. vaigiensis*. The majority of individuals recorded were smaller than 100 mm in length (Fig. 3a). Adult species were dominated by individuals from the Gobiidae and Labridae families.

### Trophic composition

**Fig. 5:**
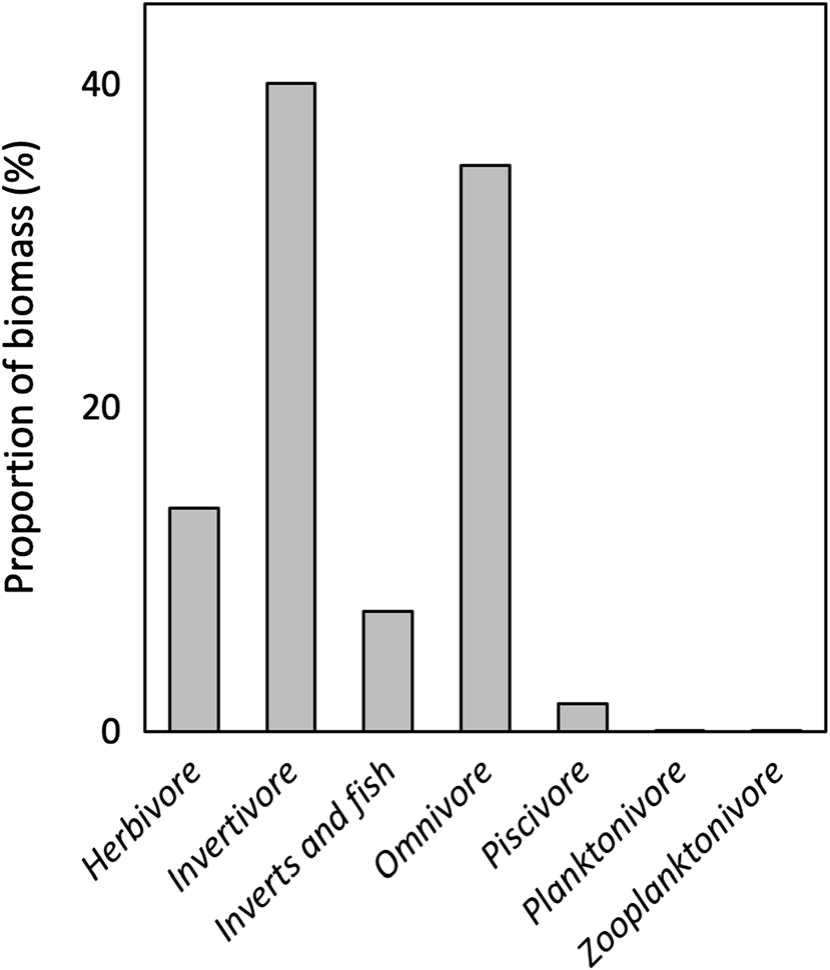
The trophic structure (percent of total weight) of drag net catches conducted over seagrass meadows in Palma Bay, Cabo Delgado Province, Mozambique

**Fig. 6:**
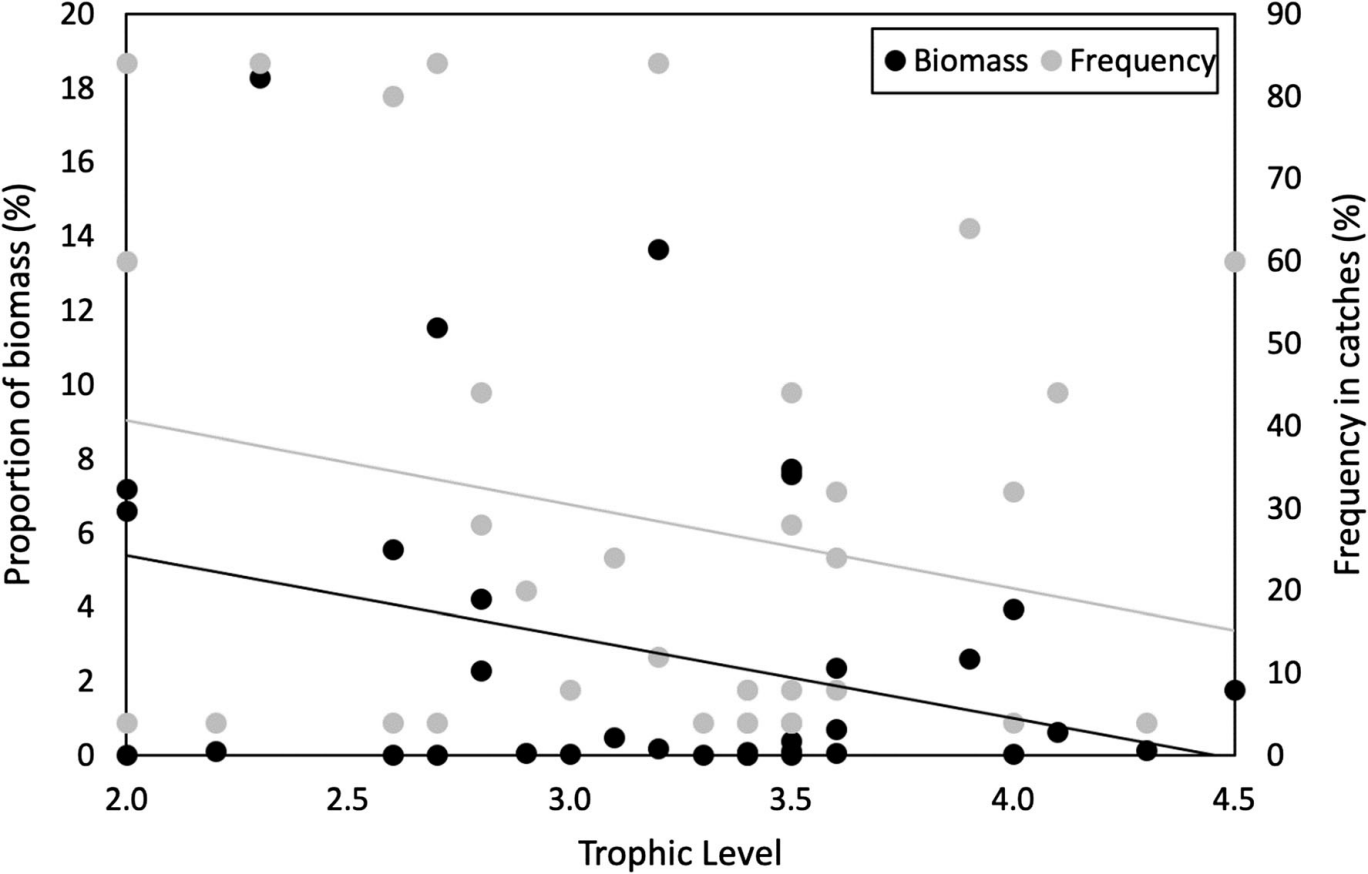
Biomass and presence of trophic levels in drag net catches conducted over seagrass meadows in Palma Bay, Cabo Delgado Province, Mozambique

Of the seven most important fish in terms of biomass (> 70%), two species were herbivores. More broadly, herbivores accounted for around 13% of the total fish biomass caught. Similarly, a low proportion of predators were recorded with the only obligate piscivore identified being *Sphyraena jello* (pickhandle barracuda). The majority of catch biomass comprised of invertivores (40%) and omnivores (35%; Fig. 5). Species at higher trophic levels were recorded in much less abundance and frequency than species at lower trophic levels (Fig. 6).

## Discussion

MNF are known to be proliferating around the tropical seas (Short et al. 2018) and their use has been argued to be unsustainable and environmentally destructive. In the present study, we provide the first empirical investigation into the sustainability of MNF by quantitatively analysing catch composition to provide evidence of the ecological cost of these fisheries. We illustrate how social and economic policies (distribution of free mosquito nets and lack of diverse livelihoods) intersect in unexpected and perverse ways that negative consequences for marine ecosystems and human well-being. Landings from these MNF may be considered unsustainable as a result of very high juvenile catch and removal of species with critical ecological functions, more so with the traditional indicators of Malthusian principles being met (Pauly 1988, 1990; Pauly et al. 1989).

The use of MNF is now accepted as a global threat (Short et al. 2018). Recent evidence of this extends throughout Asia and Africa (Srivastava et al. 2002; Abbott and Campbell 2009; Lover et al. 2011) and its use is extensive in both marine and freshwater environments. (Jiddawi and Öhman 2002; Malleret 2004; Barr 2010; Hamerlynck et al. 2011). As with other small-scale and artisanal fisheries, landings data on these fisheries are poor (Berkes 2001) and in the academic literature, we are not aware of any previous examples of the quantitative analysis of MNF landings. Due to the fine mesh size (≤ 3 mm) needed for the exclusion of mosquitos, these nets are entirely unselective if used as fishing gear. As a result, our findings provide evidence in support of previous qualitative generalisations that these MNF are of major conservation concern due to the high numbers of juveniles they catch (Short et al. 2018).

Our study revealed that across a range of sites, 56% of the species and 61% of the total biomass of catches were comprised of juvenile fish. Also, we find very low fish density when the catch is quantified per-unit-area, relative to similar regional studies with much greater diversity which we discuss further below (Gell and Whittington 2002). The levels of juvenile catch recorded for MNF are high and are comparable to other fishing gears that are being used in an unsustainable manner. A recent examination of static fish fence gears found a 40% juvenile fish count (Exton et al. 2019), whilst beach seine net gears have been recorded in Kenya to have juvenile proportions at up to 68% (Mangi and Roberts 2006).

Our study was restricted diurnally, and nocturnal fishes, particularly some of the larger predatory species that migrate into seagrass meadows during night, may not have been recorded in catches (Unsworth et al. 2007), these functionally important species were found to be in very low abundance throughout Palma Bay more broadly, indicative of a fishery in decline (Unsworth et al. 2015). Only one obligate piscivore (Pickhandle barracuda) was recorded across catches and while individuals from predatory groups such as the Snappers, Emperors and Groupers were present, these were in very low abundance in terms of frequency and biomass compared with similar and more selective regional seagrass fishery catches (Gell and Whittington 2002; de la Torre-Castro et al. 2014). From a sustainability perspective, the patterns presented here of low catch yield, lack of key functional groups and low trophic diversity likely indicate a fishery in a state of overexploitation and possibly near collapse.

The catch composition of this MNF of Mozambique indicates that this technique is far more effective in catching broad species assemblages than previously reported (Short et al. 2018). Our data record the presence of individuals from 29 families from just 25 MNF catch landings. Additionally, the average biomass for a single drag, at 1.4 kg, is high compared with the national small-scale fishery catch rate of 2.47 kg fisher^−1^ day^−1^ (Jacquet et al. 2010), especially when considering that multiple drags can be and are conducted in a single day (only 3 drags in 45 min in this study). The presence of only 39 species of marine fish and invertebrates throws into question the sustainability of MNF when compared with census data from protected seagrass meadows within the Quirimba Archipelago (< 100 km away). Using seine net catches (with similar mesh size to this study), Gell and Whittington (2002) recorded 249 species of fish in 62 families from seagrass meadows evidencing a stark difference between a more natural assemblage and the study assemblage, likely a result of intensive overfishing in the study area.

The use of mosquito nets for fishing represents a growing change demographics of the fishing community. Women (and children) contribute significantly to fisheries both directly (e.g. gleaning) and indirectly (e.g. sorting, de-scaling and selling catch), but with women and children now entering finfish fisheries (generally associated to men) and greater access to marine resources for the poor and unskilled, there is higher pressure on the marine environment. With such high juvenile catch, fishers using mosquito nets, who may already be the lowest earners of society, are putting greater pressure on their future food security. Removing fish biomass in such intensity, as reported here, can significantly alter the trophic structure of seagrass and adjacent habitats, especially when slow‐developing and economically important species are removed (Unsworth and Cullen 2010). Given that seagrass communities are defined by top-down predator control (Eklof et al. 2009; Burkholder et al. 2013), a significant loss of these predatory species can result in higher intensity (and frequency) urchin grazing events, resulting in a loss of seagrass structure and function (Eklof et al. 2008b). Coral reef fisheries provide substantial support for communities within Palma Bay. Seagrass meadows directly support coral reef productivity due to their role as feeding habitat for predatory fish and as a nursery habitat for other important reef species (Unsworth et al. 2008; Guannel et al. 2016). The removal of these species, as well as important herbivores from the Siganidae and Scaridae families, place reefs and their associated fisheries at risk (Mumby et al. 2006).

Small-scale and artisanal fisheries are dynamic (Finkbeiner 2015). They are influenced by changing drivers that present social and ecological challenges, as well as new opportunities for fishers. These opportunities are important for fishers that depend solely on such fisheries for livelihood and subsistence. The use of free or cheap mosquito nets for fishing is one such opportunity that is being harnessed globally. Here we present a unique case in which seagrass meadows and mosquito net use exists as contrasting sides of poverty alleviation (greater opportunity and empowerment yet reduced future security). Managing fisheries is intrinsically difficult when considering these fluctuating drivers (Mahon et al. 2008), and even more so with a limited understanding of how fishers and stakeholders respond to such opportunities like the distribution of mosquito nets.

In conclusion, our study presents the first quantitative analysis of marine MNF that the authors are aware of. While a small snapshot, it provides evidence of the extent of biomass removal, much of which is of juvenile fish. While mosquito nets have been distributed to improve healthcare, their use in fishing puts communities at much higher risk of future poverty due to the potential impact these gears have on the sustainability of natural resources upon which they depend. Our research highlights the need for a multi-level and cross-disciplinary approach to the management of this issue. Top-down approaches are in force that make use of mosquito nets for fishing illegal in some localities (Blythe et al. 2013), however, such mechanisms are evidently insufficient and need re-thinking. We believe that bottom-up approaches may be more beneficial to understanding the drivers that result in the use of mosquito nets in fishing, and for communities to help develop solutions to these challenges. Generally, greater effort is needed to ensure that mosquito nets are being used for the purpose intended. Such measures need to also support fisheries management initiatives in the regions in which they are used and focus on education schemes that present the issues of using mosquito nets in fisheries alongside the health issues of malaria.
